# Yeast-Produced Human Recombinant Lysosomal β-Hexosaminidase Efficiently Rescues GM2 Ganglioside Accumulation in Tay–Sachs Disease

**DOI:** 10.3390/jpm15050196

**Published:** 2025-05-10

**Authors:** Orhan Kerim Inci, Andrés Felipe Leal, Nurselin Ates, Diego A. Súarez, Angela Johana Espejo-Mojica, Carlos Javier Alméciga-Diaz, Volkan Seyrantepe

**Affiliations:** 1 Izmir Institute of Technology, Department of Molecular Biology and Genetics, Gulbahce Campus, Urla, Izmir 35430, Turkey; orhaninci@iyte.edu.tr (O.K.I.); selinates92@gmail.com (N.A.); 2 Institute for the Study of Inborn Errors of Metabolism, Faculty of Sciences, Pontificia Universidad Javeriana, Bogotá 110231, Colombia; lealb.af@javeriana.edu.co (A.F.L.); suarezd.i@javeriana.edu.co (D.A.S.); aespejo@javeriana.edu.co (A.J.E.-M.); cjalmeciga@javeriana.edu.co (C.J.A.-D.); 3 Dogma Biotech, Bogotá 110231, Colombia; 4 Izmir Institute of Technology, IYTEDEHAM, Gulbahce Campus, Urla, Izmir 35430, Turkey

**Keywords:** Tay–Sachs, murine model, GM2, rhHex-A, *P. pastoris*

## Abstract

**Background:** Tay–Sachs disease (TSD) is an autosomal recessive lysosomal storage disorder characterized by the accumulation of GM2 ganglioside due to mutations in the *HEXA* gene, which encodes the α-subunit of β-Hexosaminidase A. This accumulation leads to significant neuropathological effects and premature death in affected individuals. No effective treatments exist, but enzyme replacement therapies are under investigation. In our previous work, we demonstrated the internalization and efficacy of human recombinant lysosomal β-hexosaminidase A (rhHex-A), produced in the methylotrophic yeast *Pichia pastoris*, in reducing lipids and lysosomal mass levels in fibroblasts and neural stem cells derived from patient-induced pluripotent stem cells (iPSCs). In this study, we further evaluated the potential of rhHex-A to prevent GM2 accumulation using fibroblast and neuroglia cells from a TSD patient alongside a relevant mouse model. **Methods:** Fibroblasts and neuroglial cell lines derived from a murine model and TSD patients were treated with 100 nM rhHexA for 72 h. After treatment, cells were stained by anti-GM2 (targeting GM2 ganglioside; KM966) and anti-LAMP1 (lysosomal-associated membrane protein 1) colocalization staining and incubated with 50 nM LysoTracker Red DND-99 to label lysosomes. In addition, *GM2AP* and *HEXB* expression were analyzed to assess whether rhHex-A treatment affected the levels of enzymes involved in GM2 ganglioside degradation. **Results:** Immunofluorescence staining for LysoTracker and colocalization studies of GM2 and Lamp1 indicated reduced lysosomal mass and GM2 levels. Notably, rhHex-A treatment also affected the expression of the *HEXB* gene, which is involved in GM2 ganglioside metabolism, highlighting a potential regulatory interaction within the metabolic pathway. **Conclusions:** Here, we report that rhHex-A produced in yeast can efficiently degrade GM2 ganglioside and rescue lysosomal accumulation in TSD cells.

## 1. Introduction

GM2 gangliosidosis constitutes a cluster of lysosomal storage diseases (LSDs), encompassing Tay–Sachs disease (TSD), Sandhoff disease (SD), and GM2 activator protein deficiency (GM2AP) [1]. SD is attributed to mutations in the *HEXA* gene, which encodes the α subunit of lysosomal β-hexosaminidase A (Hex-A, EC 3.2.1.52) [2], resulting in the accumulation of GM2 ganglioside and various glycoconjugates in the central nervous system (CNS). The standard features of TSD include progressive neurodegeneration, CNS inflammation, and early death [3].

Disruption of *HEXA* in mice resulted in a near-normal phenotype rather than a severe neurological pathology, prompting the identification of a metabolic bypass in the mouse ganglioside degradation pathway. It has been suggested that sialidases are involved in the bypass pathway, catalyzing the removal of sialic acid from GM2 ganglioside to form GA2 ganglioside, which can eventually be degraded by the Hex-B enzyme in the *Hexa-/-* mouse model [4]. Consequently, severe GM2 accumulation is not evident in the TSD murine model (*Hexa-/-)*, limiting its utility as a suitable disease model. Therefore, a more suitable murine model for TSD is needed to evaluate HexA-based therapies required to treat the condition. The neuraminidase shunt pathway was recently addressed, and Neu3 was identified as the neuraminidase responsible for converting GM2 to GA2 in the *Hexa-/-* murine model. The disruption of *NEU3*, alongside *HEXA*, allowed for the generation of a novel TSD murine model (*Hexa-/-Neu3-/-*) that exhibits similar neurological symptoms to the early-onset form of TSD [5].

*Hexa-/-Neu3-/-* mice were healthy at birth but died at the age of five months due to severe neurological abnormalities, including ataxia, tremors, and weight loss. Behavioral and molecular biological analysis indicated progressively impaired motor functions and severe neurodegeneration in *Hexa-/-Neu3-/-* compared to *Hexa-/-* mice. Additionally, a massive accumulation of ganglioside GM2 has been reported in the neurons of *Hexa-/-Neu3-/-* mice. Electron and light microscopy analyses of the cortex and cerebellum in the *Hexa-/-Neu3-/-* murine model also revealed typical neuropathology similar to that observed in TSD patients [5].

In LSDs, the deficiency of specific lysosomal enzymes impairs the degradation of various macromolecules, resulting in harmful accumulation within lysosomes. In this sense, one of the main treatment alternatives for this group of disorders is enzyme replacement therapy (ERT). ERT involves the administration of recombinant enzymes specifically designed to replace deficient or absent enzymes in individuals affected by the condition. This strategy aims to restore normal enzymatic activity and reduce the accumulation of substrates that lead to cellular and organ dysfunction. Although early ERT trials aimed to correct the underlying metabolic defect by administering purified enzymes extracted from human placenta, the advent of recombinant DNA technology revolutionized the field. This advancement enabled the production of recombinant enzymes, leading to the development of ERTs now approved for the treatment of several LSDs, including Gaucher disease, Fabry disease, Pompe disease, late-infantile neuronal ceroid lipofuscinosis type II, acid lipase deficiency, alpha-mannosidosis, and mucopolysaccharidoses types I, II, IVA, VI, and VII [6,7].

Complementing these human studies, animal trials have provided valuable insights into the mechanisms of ERT. For instance, cultured cells from the Sandhoff murine model, where β-hexosaminidase deficiency leads to the accumulation of GM2 gangliosides, were treated with recombinant β-hexosaminidase-B. This resulted in substantial reductions in accumulated GM2 gangliosides, which correlated with improved neurological function in vivo [8].

Human lysosomal recombinant hexosaminidases (rhHex-A, rhHex-B, and rhHex-S) have been produced in the methylotrophic yeast *Pichia pastoris GS115* [9], and the therapeutic effects of ERT were evaluated in neural stem cells (NSCs) derived from two TS fibroblasts [10]. Additionally, in vitro evaluations in human TSD fibroblasts demonstrated that rhHex-A is taken up via mannose and mannose-6-phosphate receptors and delivered to lysosomes. It was reported that the recombinant enzyme rhHex-A could diminish stored lipids and lysosomal mass levels in human TSD fibroblasts [11]. To further evaluate the therapeutic potential of rhHex-A on TSD pathology through the ERT approach, in this study we expanded the understanding of this recombinant enzyme by (1) the use of neuroglial cells isolated from the brain of Tay–Sachs mice and neuroglial cell lines obtained from TSD patients, (2) the comparison of the efficacy of the recombinant protein in the treatment of fibroblasts obtained from the TSD mouse model and patients under the same conditions, (3) the evaluation of the effect of rhHex-A on the levels GM2 ganglioside, and (4) the evaluation of the changes in the expression of the *HEXB* gene after rhHex-A treatment.

## 2. Materials & Methods

### 2.1. Experimental Animals and Cell Culture

The animal facility laboratory at the Izmir Institute of Technology was responsible for breeding *Hexa-/-* mice in a 129/Sv background and *Neu3-/-* mice in a C57BL/6 background to generate *wild-type (WT)*, *Hexa-/-*, and *Hexa-/-Neu3-/-* mouse models. The procedures for the care and use of mice are based on the Institutional Animal Care and Use Committee of Izmir Institute of Technology guidelines. Genotyping was performed by PCR using genomic DNA from the mice’s tails. The *WT* and Hexa alleles were amplified by using the primers Hexa F (5′-GGCCAGATACAATCATACAG-3′), Hexa R (5′-CTGTCCACATACTCTCCCCACAT-3′), and PGK-R (5′-CACCAAAGAAGGGAGC CGGT-3′). In addition, *WT* and Neu3 alleles were amplified by using the following primers: Neu3 F (5′-GCTCTACCCCATTCTACATCTCCAGAC-3′), Neu3 R (5′-TCGT GCTTTACGGTATCGCCGCTCCCGATT-3′), and Neo-R (5′-GTGAGTTCAAGAGC CA TGTTGCTGATGGTG-3′).

Mouse fibroblasts were derived from skin biopsies taken from 5-month-old *WT*, *Hexa-/-*, and *Hexa-/-Neu3-/-* mice. The fibroblasts were cultured in a humidified incubator at 37 °C with 5% CO_2_ in Dulbecco’s Modified Eagle’s Medium (DMEM) (Gibco, Thermo Fisher Scientific, Waltham, MA, USA) supplemented with 10% fetal bovine serum (FBS) (Gibco, Thermo Fisher Scientific, Waltham, MA, USA) and 1% penicillin/streptomycin (Gibco, Thermo Fisher Scientific, Waltham, MA, USA). Additionally, we received human control and TS fibroblast samples as a generous gift from Dr. Nur Arslan in the Department of Child Neurology at Dokuz Eylül University.

For immortalization, primary fibroblast lines were treated with a recombinant retrovirus expressing the HPV16 E6 and E7 genes (LXSN 16E6E7) and polybrene (400 µg/mL) in serum-free DMEM overnight at 37 °C. The next day, the viral supernatant and DMEM supplemented with polybrene were removed, and the cells were washed with PBS. After that, cells were grown for 10 days in DMEM containing 10% FBS and G418 (400 µg/mL) to ensure the selection of infected cells.

Neuroglia lines were established from *WT*, *Hexa-/-*, and *Hexa-/-Neu3-/-* mice using 100 mg of brain tissue obtained from 5-month-old mice of each genotype, following the manufacturer’s instructions (Miltenyi Biotec, Bergisch Gladbach, Germany, 130-107-677) for the adult brain dissociation kit. Following removing cellular debris and red blood cells, neurons were isolated using a neuron isolation kit according to the manufacturer’s guidelines (Miltenyi Biotec, Bergisch Gladbach, Germany, 130-115-390). The cells were subsequently cultured in a humidified incubator set at 37 °C with 5% CO_2_, using DMEM (Gibco, Thermo Fisher Scientific, Massachusetts, USA) supplemented with 10% fetal bovine serum (Gibco, Thermo Fisher Scientific, Massachusetts, USA) and 1% penicillin/streptomycin (Gibco, Thermo Fisher Scientific, Massachusetts, USA).

The immortalized human neuroglia cells (NG125) from TS patients [12] and control human neuroglia cells (NG124) containing cDNA encoding the α subunit of Hex A (pCMV-Hex α), which produces active HexA enzyme [13], were cultured in Dulbecco’s Modified Eagle’s Medium (DMEM) containing 10% FBS and 1% penicillin/streptomycin.

### 2.2. Recombinant β-Hexosaminidases a Production

Recombinant β-hexosaminidase A was produced from a previously selected *P. pastoris* GS115 clone [9]. Cultures were prepared in a modified FM22 saline medium (composition per liter: 25.74 g KH_2_PO_4_, 3 g (NH_4_)_2_SO_4_, 8.58 g K_2_SO_4_, 0.6 g CaSO_4_ 2H_2_O, 40 g glycerol, 7.02 g MgSO_4_ 7H2O, 4 × 10^−5^% biotin, 1 mL mineral traces, and 5 mL silicone). Protein production was carried out in three phases: (i) a batch culture with glycerol to achieve 40 g L^−1^ biomass, (ii) a fed-batch culture with glycerol to obtain 60 g.L^−1^ biomass, and (iii) a fed-batch induction phase with methanol concentration maintained at 0.5 ± 0.005% with an automatic feed control. The cultures were maintained at pH 5.0 (adjusted with 7% ammonium hydroxide), 28 °C, and with limited oxygen [9]. As previously described, the recombinant enzyme was purified by slightly modifying the standardized protocol of [9]. The filtered crude extract was briefly diafiltered against 10 mM phosphate buffer, pH 6.0, and purified by anion exchange chromatography (Hi Trap DEAE FF, Amersham, United Kingdom) on an AKTA Pure chromatograph. The eluted fractions were pooled, concentrated, and then diafiltered through a 30 kDa membrane against stability buffer (25 mM citrate buffer, pH 4.5). The production and purification processes of rhHex-A were followed by enzyme activity assays using 4-methylumbelliferyl-β-D-acetyl-glucosaminide (MUG) or 4-methylumbelliferyl-β-D-acetyl-glucosaminide sulfate (MUGS) substrates [14] and by SDS-PAGE.

### 2.3. rhHex-A Treatment

Fibroblast and neuroglia cell lines derived from the murine model and TSD patients were grown on 24-well plates for immunocytochemistry and 100 mm culture disks for gene expression analysis. Before experiments, cells were treated with 100 nM rhHex-A and incubated for 72 h [11]. An MTT (3-[4,5-dimethylthiazol-2-yl]-2,5 2,5-diphenyl tetrazolium bromide) assay for cell viability was performed for WT fibroblasts and neuroglia cells to determine the optimal concentration of rhHex-A treatment. It has been shown that the recombinant protein applied at different concentrations for 72 h in fibroblast and neuroglia cells causes toxicity in these cells at concentrations of 150 nM and above. Therefore, the optimum enzyme concentration was 100 nM (Figure S1).

### 2.4. q-RT PCR Analysis

Total RNA was extracted from mouse fibroblasts (n = 3) using Trizol reagent (Geneaid, New Taipei City, Taiwan). The following cDNAs were then synthesized using a reverse transcription kit (BioRad, Hercules, CA, USA) according to the manufacturer’s instructions. Relative mRNA expression analysis of the genes involved in GM2 ganglioside degradation was carried out; *HexB* and *GM2AP* were analyzed by a Roche Light Cycler 96 machine using Real-Time SYBER green PCR master mix (Roche, Basel, Switzerland) with the following conditions: initial denaturation at 95 °C for 10 min; 45 cycles at 95 °C for 20 s and 61 °C for 15 s. GAPDH gene expression was employed as an endogenous control. The primers used for expression analysis are listed in Table 1.

### 2.5. Immunocytochemistry Analysis

The verification that the rhHex-A enzyme reduces the lysosomal mass was demonstrated by LysoTracker staining. Control and TS fibroblasts, as well as mouse *Hexa-/-* and *Hexa-/-Neu3-/-* fibroblast cell lines, were used, along with human neuroglia NG124 and NG125 and mouse neuroglia *WT*, *Hexa-/-*, and *Hexa-/-Neu3-/-* cell lines. The cells were grown on coverslips placed onto 24-well plates and treated with rhHex-A (100 nM) for 72 h. After 72 h of incubation, the cells were incubated with LysoTracker Red (50 nM) for 1 h and then fixed with 4% PFA (paraformaldehyde) for 30 min. To determine whether rhHex-A reduces GM2 ganglioside accumulation in lysosomes, co-staining with anti-GM2 and anti-LAMP1 (lysosomal-associated membrane protein 1) was performed for each cell. The cells were grown on 24-well plates and treated with rhHex-A (100 nM) for 72 h. The antibodies targeting LAMP1 and GM2 (GM2 ganglioside; KM966) were diluted to 1:500 in the blocking solution and applied overnight at 4 °C. Goat anti-human DyLight 488 (Thermo Fisher Scientific, Massachusetts, USA) and goat anti-rabbit Alexa Fluor 568 (Abcam, Cambridge, United Kingdom) fluorescent antibodies were used to observe the binding of anti-GM2 and anti-LAMP1 antibodies, respectively. The slides were mounted with Fluoroshield mounting medium with DAPI (Abcam, Cambridge, United Kingdom), followed by fluorescence microscopy imaging (Olympus, Hamburg, Germany).

### 2.6. Statistical Analysis

All statistical analyses were performed using GraphPad Prism 7. In vitro, human control and patient fibroblasts and neuroglial cells and *WT*, *Hexa-/-*, and *Hexa-/-Neu3-/-* mouse fibroblasts and neuroglial cells were compared using one-way ANOVA. All data are displayed as mean ± SEM.

## 3. Results

### 3.1. Human Recombinant Hex-A (rhHex-A) Treatment Significantly Reduces Lysosomal Mass in TS Cells

The human recombinant Hex-A was produced in *P. pastoris* using a previously selected producer clone [9]. For this study, rhHex-A showed an activity of 143 nmol/h/mg and an expected SDS-PAGE profile with no other evident proteins (Figure S2). Previously, it was shown that human recombinant lysosomal enzymes produced in *P. pastoris*, including rhHex-A, were efficiently taken up at a 50 to 100 nM concentration, and that higher concentrations did not lead to an efficient process [15,16,17]. In addition, the optimal enzyme concentration used in this study was established to be 100 nM according to MTT permeability assays, since higher concentrations caused cell toxicity.

**Figure 1:**
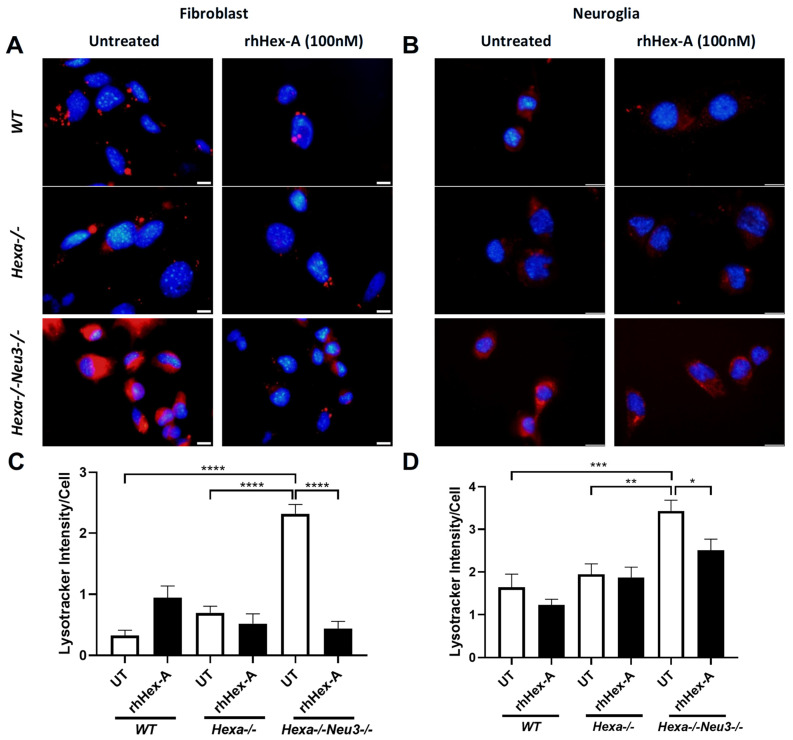
LysoTracker staining of recombinant human Hexa protein (rhHex-A, 100 nM) in treated fibroblasts (**A**) and neuroglia (**B**) derived from wild-type *(WT)*, *Hexa-/-*, and *Hexa-/-Neu3-/-* mice. ImageJ v1.54 program measured the total LysoTracker signal, and the histogram for LysoTracker intensity per cell for fibroblasts (**C**) and neuroglia (**D**) is represented. Images were taken under the same light intensity at 100× magnification, and the data are presented as the mean ± S.E.M. Two-way ANOVA analysis was used to determine *p*-values, as indicated by GraphPad (* *p* < 0.05, ** *p* < 0.01, *** *p* < 0.005, and **** *p* < 0.001) (n = 3).

**Figure 2:**
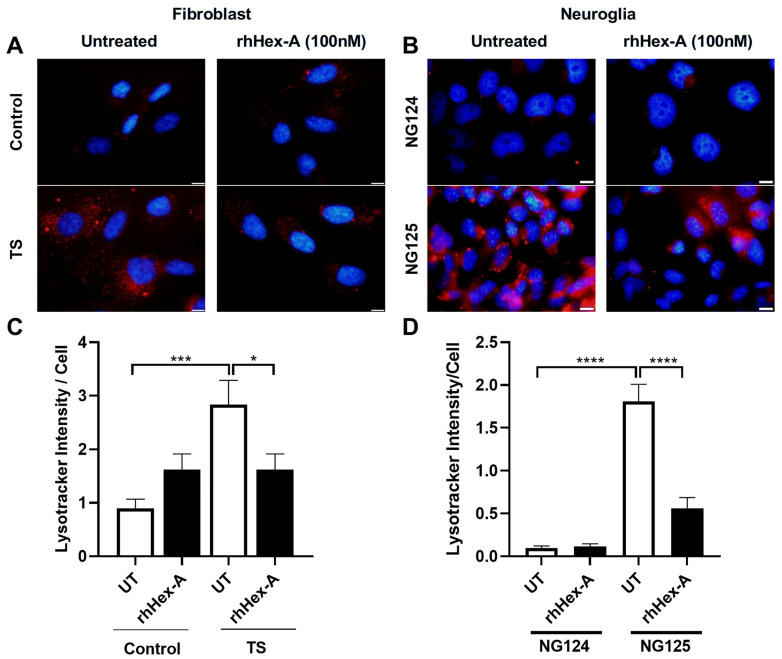
LysoTracker staining of recombinant human Hexa protein (rhHex-A, 100 nM)-treated fibroblasts (**A**) and neuroglia (**B**) cells derived from a human TS patient. ImageJ v1.54 program was used to measure the total LysoTracker signal, and a histogram representing the LysoTracker intensity per cell was generated (**C**,**D**). Images were taken under the same light intensity at 100× magnification, and the data are presented as the mean ± S.E.M. Two-way ANOVA analysis was used to determine *p*-values, as indicated by GraphPad (* *p* < 0.05, *** *p* < 0.005, and **** *p* < 0.001) (n = 3).

The effect of rhHex-A on lysosomal storage was studied in fibroblasts and neuroglia cells derived from the TSD murine model (*Hexa-/-Neu3-/-*) and TSD patients. The fibroblasts were treated with 100 nM rhHex-A for 72 h and then stained with LysoTracker Red, which allows for labeling acidic compartments. As observed in Figure 1A, the TSD mouse fibroblast showed a significant reduction in lysosomal mass after rhHex-A treatment compared to the untreated cells (Figure 1A,C). When neuroglia cells were examined, as in mouse fibroblasts, a significant increase in LysoTracker concentrations was observed in the TSD mouse model compared to neuroglia cell lines generated from age-matched *WT* and *Hexa-/-* mice. Lysosomal mass in neuroglia cells was significantly decreased after 72 h of rhHex-A treatment (Figure 1B,D). Similarly, the rhHex-A treatment decreased LysoTracker intensity in human TS fibroblasts, leading to normalization of the lysosomal storage compared to control cells (Figure 2A,C). These findings suggest that TSD fibroblasts derived from mice and patients have an elevated lysosomal mass that can be both reduced and returned to a normal level after treatment with rhHex-A. In addition, we confirmed that, as observed in fibroblasts, treatment of NG125 with rhHex-A resulted in a significant decrease in LysoTracker Red intensity (Figure 2B,D).

### 3.2. Abnormal GM2 Accumulation Was Mitigated by rhHex-A Treatment in TS Cells

**Figure 3:**
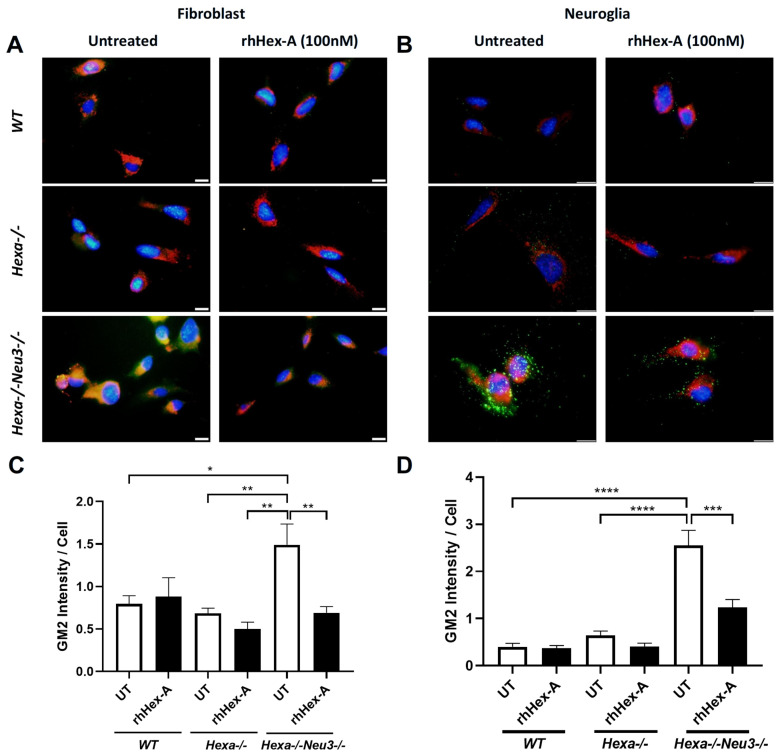
GM2/Lamp-1 staining of recombinant human Hexa protein (rhHex-A, 100 nM)-treated fibroblasts (**A**) and neuroglia cells (**B**) from *WT*, *Hexa-/-* and *Hexa-/-Neu3-/-* mice. Total GM2 signal was measured by ImageJ v1.54 program and histograms for GM2 intensity per cell for fibroblasts (**C**) and neuroglia (**D**) are represented. Images were taken under the same light intensity at 100× magnification, and the data are represented as the mean ± S.E.M. Two-way ANOVA analysis was used to determine *p*-values, as indicated by GraphPad (* *p* < 0.05, ** *p* < 0.01, *** *p* < 0.005, and **** *p* < 0.001) (n = 3).

**Figure 4:**
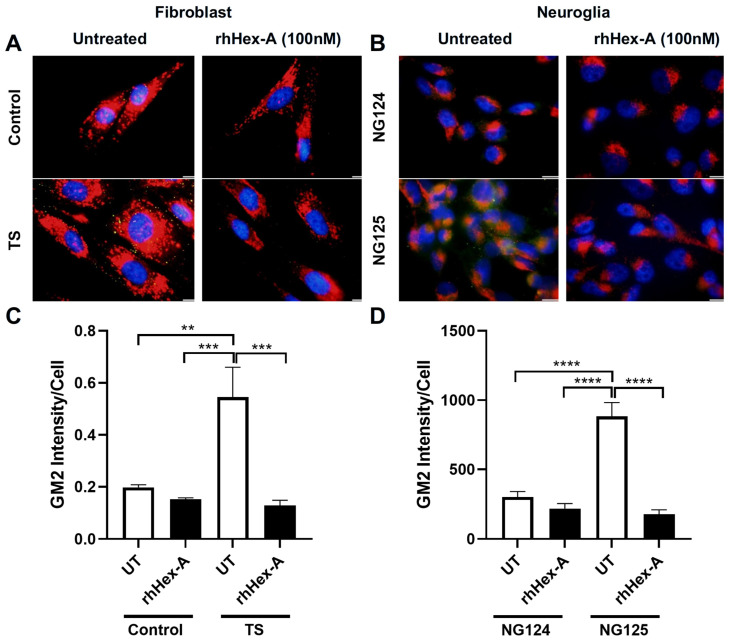
GM2/Lamp-1 staining of recombinant human Hexa protein (rhHex-A, 100 nM)-treated fibroblasts (**A**) and neuroglia (**B**) cells derived from a human TSD patient. ImageJ v1.54 program was used to measure the total GM2 signal, and histograms representing the GM2 intensity per cell for fibroblasts (**C**) and neuroglia (**D**) are shown. Images were taken under the same light intensity at 100× magnification, and the data are presented as the mean ± S.E.M. Two-way ANOVA analysis was used to determine *p*-values, as indicated by GraphPad (** *p* < 0.01, *** *p* < 0.005, and **** *p* < 0.001) (n = 3).

GM2 ganglioside accumulation is the most prominent pathological evidence in cells from TS patients and the *Hexa-/-Neu3-/-* murine model. Immunocytochemistry analysis was performed on GM2 ganglioside after treating TSD cells with 100 nM rhHex-A for 72 h to evaluate the potential therapeutic effect of the recombinant protein on lysosomal GM2 ganglioside accumulation. Untreated fibroblasts (Figure 3A) and neuroglia cells (Figure 3B) from *Hexa-/-Neu3-/-* mice displayed a significant increase in GM2 ganglioside compared to *WT* and *Hexa-/-* cells. Notably, the accumulation of GM2 ganglioside in *Hexa-/-Neu3-/-* fibroblasts was significantly reduced after rhHex-A treatment compared to the untreated group. However, WT and *Hexa-/-* fibroblasts were not affected by rhHex-A treatment (Figure 3A,C). We observed a significant decrease in the amount of GM2 ganglioside in mouse neuroglia cells after treatment (Figure 3B,D). Similarly, we observed that rhHex-A treatment reversed GM2 accumulation in both patient fibroblasts (Figure 4A,C) and neuroglia cells (Figure 4B,D) compared to untreated conditions.

### 3.3. The Elevated Expression Level of HexB Gene After rhHex-A Treatment in Hexa-/-Neu3-/- Fibroblasts

**Figure 5:**
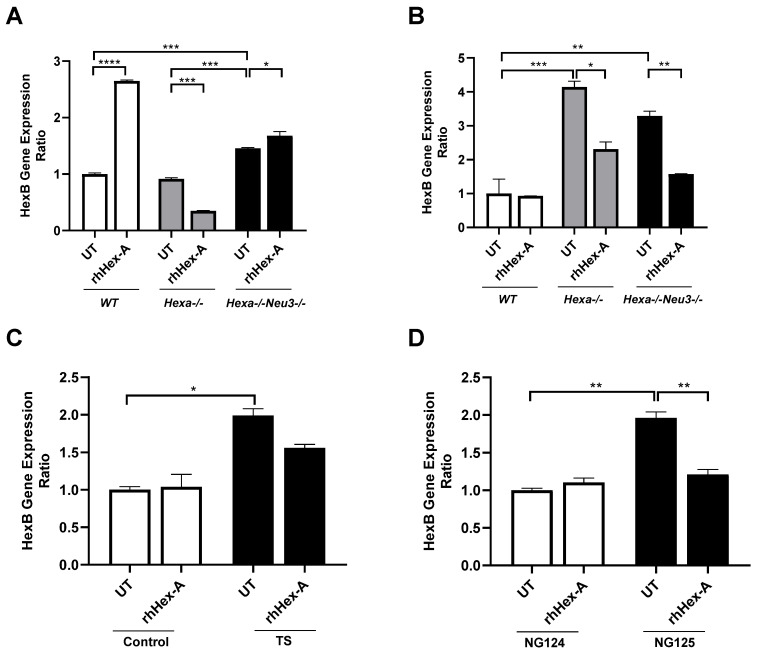
*HexB* gene expression analysis in mouse fibroblast (**A**) and neuroglia (**B**) generated from *WT*, *Hexa-/-*, and *Hexa-/-Neu3-/-* mice as well as human fibroblasts (**C**) and neuroglia (**D**) in untreated (UT) and recombinant human HexA protein (rhHex-A, 100 nM for 72 h)-treated conditions. The data are represented as the mean ± S.E.M. Two-way ANOVA analysis was used to determine *p*-values, as shown in GraphPad (* *p* < 0.05, ** *p* < 0.01, *** *p* < 0.005, and **** *p* < 0.001).

**Figure 6:**
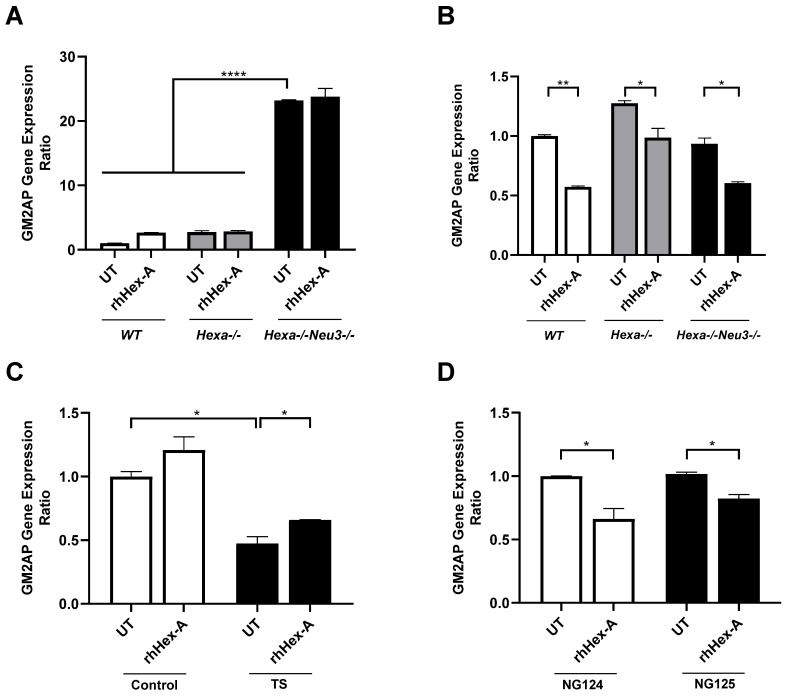
*GM2AP* gene expression analysis in mouse fibroblasts (**A**) and neuroglia (**B**) generated from *WT*, *Hexa-/-*, and *Hexa-/-Neu3-/-* mice as well as human fibroblasts (**C**) and neuroglia (**D**) in untreated (UT) and recombinant human Hexa protein (rhHex-A, 100 nM for 72 h)-treated conditions. The data are represented as the mean ± S.E.M. Two-way ANOVA analysis was used to determine *p*-values, as shown in GraphPad (* *p* < 0.05, ** *p* < 0.01, and **** *p* < 0.001).

Previously, it was demonstrated that TSD patients and *Hexa-/-Neu3-/-* fibroblasts exhibit elevated levels of HEXA-related gene expression (HEXB and GM2AP) compared to WT and *Hexa-/-* fibroblasts (Seyrantepe et al., 2018). Here, it was demonstrated that the level of *HexB* expression in *wild-type* (WT) mouse fibroblasts was significantly higher after rhHex-A treatment. Although the level of *HexB* expression is higher in *Hexa-/-Neu3-/-* fibroblasts compared to *WT* and *Hexa-/-*, only a slight elevation is detected after treatment (Figure 5A). In untreated mouse neuroglia cells, the level of *HexB* expression was significantly higher in *Hexa-/-* and *Hexa-/-Neu3-/-* mice compared to WT mice. rhHex-A treatment resulted in a reduction in the level of *HexB* expression in both *Hexa-/-* and *Hexa-/-Neu3-/-* neuroglial cells (Figure 5B). In parallel, both TSD fibroblasts and TSD neuroglia cells displayed an elevated level of *HexB* expression, which was significantly reduced only in NG125 cells after rhHex-A treatment (Figure 5C,D). Consistent with our previous findings in the *Hexa-/-Neu3-/-* cortex [5], the level of *Gm2ap* expression was found to be significantly elevated in untreated *Hexa-/-Neu3-/-* fibroblasts (Figure 6A). However, this increase was not significant in untreated *Hexa-/-* and *Hexa-/-Neu3-/-* neuroglial cells compared to *WT* (Figure 6B). Moreover, the level of *Gm2ap* expression was slightly reduced following rhHex-A treatment, particularly in all neuroglial cells derived from mice (Figure 6B). Surprisingly, the expression level did not change in *Hexa-/-Neu3-/-* fibroblasts after treatment (Figure 6A). A lower level of *GM2AP* expression in untreated human TS fibroblasts was detected, possibly related to a mutation in the *HEXA* gene and impaired protein degradation machinery. The increase in *GM2AP* expression levels may be due to the high amount of recombinant enzyme required in TSD fibroblasts to degrade accumulated GM2 gangliosides (Figure 6C). Similarly to the response in mouse neuroglia, the level of *GM2AP* expression in human neuroglial cells decreased after treatment (Figure 6D).

## 4. Discussion

TSD is a severe neurodegenerative disorder characterized by the progressive lysosomal accumulation of GM2 in neurons due to a deficiency of the HexA enzyme, which results in early death of patients [18].

The complex clinical phenotypes of neurodegenerative LSDs pose significant challenges for the development of treatments, as therapeutics often fail to cross the blood–brain barrier (BBB) [19]. Despite this, ongoing in vitro and in vivo studies continue to explore the efficacy of ERT, which has been particularly effective in treating Gaucher, Fabry, and Pompe diseases [20,21,22]. To enhance the targeted delivery of recombinant enzymes to the brain, direct administration through intrathecal (IT) or intracerebroventricular (ICV) methods, which completely bypass the BBB, has been explored [23]. For example, the recombinant human tripeptidyl peptidase 1 enzyme (cerliponase alfa) has been delivered as the first approved therapy via ICV infusion to treat patients with neuronal ceroid lipofuscinosis type 2 (CLN2) [24]. Furthermore, for Sanfilippo type B (MPS type IIIB) patients with NAGLU mutations, who were treated intracerebroventricularly with tralesinidaz alfa as part of phase I/II studies, the level of heparan sulfate accumulated in these patients returned to normal as a result of treatment [25].

Successful therapy development requires a deep understanding of the biochemical properties of therapeutic enzymes and the distribution of storage products in various tissues, as well as insights into disease mechanisms and the use of effective testing models. To improve the efficacy of ERT, researchers have explored various expression systems for producing enzymes with glycosylation patterns similar to those found in humans. Chinese hamster ovary (CHO) cells are commonly used because they can perform complex glycosylation essential for enzyme function and stability [8,26]. Preclinical assays using modified recombinant β-Hexosaminidase B produced in CHO cells, administered ICV in Sandhoff disease murine models, showed significant reductions in ganglioside accumulation in the parenchyma [8]. Human epithelial cells, such as HeLa cells, also provide human-like glycosylation patterns that may enhance enzyme uptake and efficacy [27]. Insect cell systems, such as those derived from Sf9 cells, and yeast systems, particularly *Ogataea minuta* and *Pichia pastoris*, have garnered attention for their favorable glycosylation and efficient secretion mechanisms [9,28,29]. A hypermannosylated recombinant Hex-A produced in *O. minuta* resulted in a 7.8% increase in life expectancy in the Sandhoff murine model [29].

Notably, *P. pastoris* allows for high yields of recombinant proteins, which is crucial for large-scale ERT applications. *P. pastoris* has also been utilized to develop recombinant enzymes, including β-hexosaminidases, to mitigate accumulated GM2 [9]. Recently, rhHex-A produced in *P. pastoris*, without any modification of the N-glycan content, showed comparable efficacy with enhanced enzyme uptake [9]. We confirmed that the cell uptake of this rhHex-A was an endocytosis-mediated process [11]. This was performed by evaluating the enzyme uptake at 37 °C and 4 °C, showing that the recombinant protein was significantly internalized at 37 °C, but not at 4 °C. Then, the receptors used by rhHexA to be taken up by human skin fibroblasts through a cell uptake assay in the presence or absence of mannose or mannose-6-phosphate (M6P) were evaluated [11]. The results showed that rhHexA uptake was inhibited in the presence of both mannose and M6P, suggesting a mannose and M6P receptor-mediated internalization of this recombinant enzyme. In addition, this cellular uptake process has also been observed for other recombinant lysosomal enzymes produced in *Pichia pastoris* under the same conditions as rhHexA, such as GALNS [15], IDS [17], and NAGLU [30].

In general, despite significant advancements in understanding the pathophysiology of LSDs, the urgent need for effective curative treatments remains critical. In this proof-of-concept study, we evaluated the therapeutic potential of rhHex-A produced in *P. pastoris* in reverting the TSD phenotype through an in vitro ERT approach. We extended our work by demonstrating that rhHex-A can reduce lysosomal mass and lipids in fibroblasts and neuroglial cell lines derived from a novel TSD mouse model and TS patients. For the first time, we showed the results of in vitro treatment with *P. pastoris*-derived rhHex-A on TS neuroglia cells. We demonstrated that cells effectively took up the rhHex-A, which reduced abnormal GM2 ganglioside accumulation and lysosomal mass with no signs of associated toxicity, suggesting the potential for similar outcomes in in vivo conditions.

HEXB is the beta subunit of the lysosomal enzyme beta-hexosaminidase. Along with GM2AP as a cofactor, it plays a crucial role in breaking down GM2 ganglioside [31]. We also examined whether rhHex-A treatment affected the expression levels of these HexA-associated genes. Consistent with our previous findings, *Hexb* expression was significantly elevated in *Hexa-/-Neu3-/-* cells under untreated conditions, likely reflecting a compensatory mechanism for enzyme deficiencies [5]. Treatment with rhHex-A resulted in a notable reduction in *HexB* expression in both human and mouse neuroglial cells. Similarly, analysis of *GM2AP* gene expression levels in neuroglial cells revealed a decrease following treatment with rhHex-A. These findings align with our immunocytochemical results, suggesting that rhHex-A treatment is more effective in reducing GM2 accumulation in neuroglial cell lines, which may result from the more severe pathology observed in these cell lines. The reduction in the expression of Hexa-related genes is possibly due to compensatory mechanisms within the ganglioside biosynthesis and degradation pathways in response to rhHex-A treatment. The differences observed between cell types may result from variations in residual enzyme activity among the different cell types. Furthermore, the rhHex-A enzyme activity level might vary depending on the specific cell type and organism.

Overall, our results provide compelling evidence for the importance of *P. pastoris* as an expression host for producing recombinant lysosomal enzymes, with potential applications for ERT in TSD. We suggest that the delivery of rhHex-A to the central nervous system (CNS) when administered through the intracerebroventricular (ICV) route may reduce neuronal GM2 accumulation in the *Hexa-/-Neu3-/-* mouse model.

This ERT approach using rhHex-A produced in yeast could represent a cost-effective strategy for treating TSD. It may also pave the way for novel therapeutic interventions for other LSDs where there is CNS involvement.

## 5. Conclusions

Tay–Sachs neuroglia and fibroblast cells from mice and humans were treated with human recombinant Hex-A protein, purified from the methylotrophic yeast *P. pastoris*. Treatment with rhHex-A reduced GM2 ganglioside accumulation and altered the levels of HEXA-related gene (*HEXB* and GM2AP) expression in these cells. Based on the in vitro results, rhHex-A could potentially be developed for further in vivo studies in the Tay–Sachs mouse model.

## Figures and Tables

**Table 1:** Primer sequences of mouse genes used in q-PCR analysis.

mHexB	F: 5′-AGTGCGAGTCCTTCCCTAGT-3′ R: 5′-ATCCGGACATCGTTTGGTGT-3′	412 bp
mGM2AP	F: 5′-GCTGGCTTCTGGGTCAAGAT-3′ R: 5′-GCACTGTGAAGTTGCTCGTG-3′	193 bp
mGAPDH	F: 5′-CCCCTTCATTGACCTCAACTAC-3′ R: 5′-ATGCATTGCTGACAATCTTGAG-3′	347 bp
hHexB	F: 5′-GTTTTGGATATTATTGCAACCATAAA-3′ R: 5′-AGTACAGATTGCTGTGGCCT-3′	338 bp
hGM2AP	F: 5′-TACCTATGGGCTTCCTTGCCAC-3′ R: 5′-GACGCTCTCTATGCGGTAGTTC-3′	130 bp
hGAPDH	F: 5′-CCCCTTCATTGACCTCAACTAC-3′ R: 5′-ATGCATTGCTGACAATCTTGAG-3′	347 bp

## Data Availability

The data are included in the manuscript.

## Supplementary Materials

The following supporting information can be downloaded at: https://www.mdpi.com/article/10.3390/jpm15050196/s1, Figure S1: Cell viability assessed by MTT assay. WT fibroblasts (A) and neuroglia (B) cells were treated with 5 nM, 25 nM, 100 nM, 150 nM and 250 nM at the indicated doses for 72 hours. The data are represented as the mean ± S.E.M. One-way ANOVA analysis was used to determine p-values, as GraphPad shows.; Figure S2: SDS-PAGE of rhHexA purification. The eluted fractions (5 to 13) obtained during ion exchange chromatography of rhHexA were analyzed by SDS-PAGE under reducing conditions. MW: molecular weight.

## References

[1] Platt F.M., Boland B., van der Spoel A.C. (2012). The cell biology of disease: Lysosomal storage disorders: The cellular impact of lysosomal dysfunction. J. Cell Biol..

[2] Breiden B., Sandhoff K. (2019). Lysosomal Glycosphingolipid Storage Diseases. Annu. Rev. Biochem..

[3] Beck M., Clarke J.T.R., Sandhoff K., Mehta A.B., Winchester B. (2022). The Gangliosidoses. Lysosomal Storage Disorders.

[4] Yuziuk J.A., Bertoni C., Beccari T., Orlacchio A., Wu Y.Y., Li S.C., Li Y.T. (1998). Specificity of mouse GM2 activator protein and beta-N-acetylhexosaminidases A and B. Similarities and differences with their human counterparts in the catabolism of GM2. J. Biol. Chem..

[5] Seyrantepe V., Demir S.A., Timur Z.K., Von Gerichten J., Marsching C., Erdemli E., Oztas E., Takahashi K., Yamaguchi K., Ates N. (2018). Murine Sialidase Neu3 facilitates GM2 degradation and bypass in mouse models of Tay-Sachs disease. Exp. Neurol..

[6] Sun A. (2018). Lysosomal storage disease overview. Ann. Transl. Med..

[7] Desnick R.J., Schuchman E.H. (2012). Enzyme replacement therapy for lysosomal diseases: Lessons from 20 years of experience and remaining challenges. Annu. Rev. Genom. Hum. Genet..

[8] Matsuoka K., Tamura T., Tsuji D., Dohzono Y., Kitakaze K., Ohno K., Saito S., Sakuraba H., Itoh K. (2011). Therapeutic potential of intracerebroventricular replacement of modified human β-hexosaminidase B for GM2 gangliosidosis. Mol. Ther. J. Am. Soc. Gene Ther..

[9] Espejo Mojica A.J., Mosquera A., Rodríguez-López A., Díaz D., Beltrán L., Hernández F.L., Alméciga Díaz C.J., Barrera L.A. (2016). Characterization of recombinant human lysosomal beta-hexosaminidases produced in the methylotrophic yeast Pichia pastoris. Univ. Sci..

[10] Vu M., Li R., Baskfield A., Lu B., Farkhondeh A., Gorshkov K., Motabar O., Beers J., Chen G., Zou J. (2018). Neural stem cells for disease modeling and evaluation of therapeutics for Tay-Sachs disease. Orphanet J. Rare Dis..

[11] Espejo-Mojica A.J., Rodríguez-López A., Li R., Zheng W., Alméciga-Díaz C.J., Dulcey-Sepúlveda C., Combariza G., Barrera L.A. (2020). Human recombinant lysosomal β-Hexosaminidases produced in Pichia pastoris efficiently reduced lipid accumulation in Tay-Sachs fibroblasts. Am. J. Med. genetics Part C Semin. Med. Genet..

[12] Hoffman L.M., Amsterdam D., Schneck L. (1976). GM2 ganglioside in fetal Tay-Sachs disease brain cultures: A model system for the disease. Brain Res..

[13] Fernandes M.J., Yew S., Leclerc D., Henrissat B., Vorgias C.E., Gravel R.A., Hechtman P., Kaplan F. (1997). Identification of candidate active site residues in lysosomal beta-hexosaminidase A. J. Biol. Chem..

[14] Shapira E., Blitzer M.G., Miller J.B., Africk D.K. (1989). Biochemical Genetics: A Laboratory Manual.

[15] Rodríguez-López A., Alméciga-Díaz C.J., Sánchez J., Moreno J., Beltran L., Díaz D., Pardo A., Ramírez A.M., Espejo-Mojica A.J., Pimentel L. (2016). Recombinant human N-acetylgalactosamine-6-sulfate sulfatase (GALNS) was produced in the methylotrophic yeast Pichia pastoris. Sci. Rep..

[16] Rodríguez-López A., Pimentel-Vera L.N., Espejo-Mojica A.J., Van Hecke A., Tiels P., Tomatsu S., Callewaert N., Alméciga-Díaz C.J. (2019). Characterization of Human Recombinant N-Acetylgalactosamine-6-Sulfate Sulfatase Produced in Pichia pastoris as a Potential Enzyme for Mucopolysaccharidosis IVA Treatment. J. Pharm. Sci..

[17] Pimentel N., Rodríguez-Lopez A., Díaz S., Losada J.C., Díaz-Rincón D.J., Cardona C., Espejo-Mojica Á.J., Ramírez A.M., Ruiz F., Landázuri P. (2018). Production and characterization of a human lysosomal recombinant iduronate-2-sulfatase produced in Pichia pastoris. Biotechnol. Appl. Biochem..

[18] Cachon-Gonzalez M.B., Zaccariotto E., Cox T.M. (2018). Genetics and Therapies for GM2 Gangliosidosis. Curr. Gene Ther..

[19] Begley D.J., Bellettato C.M., Scarpa M., Mehta A.B., Winchester B. (2022). Central nervous system aspects, neurodegeneration, and the blood-brain barrier. Enzyme Replacement Therapies in Neurodegenerative Disorders.

[20] Sun Y., Liou B., Chu Z., Fannin V., Blackwood R., Peng Y., Grabowski G.A., Davis H.W., Qi X. (2020). Systemic enzyme delivery by blood-brain barrier-penetrating SapC-DOPS nanovesicles for treatment of neuronopathic Gaucher disease. EBioMedicine.

[21] Fellgiebel A., Gartenschläger M., Wildberger K., Scheurich A., Desnick R.J., Sims K. (2014). Enzyme replacement therapy stabilized white matter lesion progression in Fabry disease. Cerebrovasc. Dis..

[22] Chien Y.H., Tsai W.H., Chang C.L., Chiu P.C., Chou Y.Y., Tsai F.J., Wong S.L., Lee N.C., Hwu W.L. (2020). Earlier and higher dosing of alglucosidase alfa improves outcomes in patients with infantile-onset Pompe disease: Evidence from real-world experiences. Mol. Genet. Metab. Rep..

[23] Edelmann M.J., Maegawa G.H.B. (2020). CNS-Targeting Therapies for Lysosomal Storage Diseases: Current Advances and Challenges. Front. Mol. Biosci..

[24] de Los Reyes E., Lehwald L., Augustine E.F., Berry-Kravis E., Butler K., Cormier N., Demarest S., Lu S., Madden J., Olaya J. (2020). Intracerebroventricular Cerliponase Alfa for Neuronal Ceroid Lipofuscinosis Type 2 Disease: Clinical Practice Considerations From US Clinics. Pediatr. Neurol..

[25] Muschol N., Koehn A., von Cossel K., Okur I., Ezgu F., Harmatz P., de Castro Lopez M.J., Couce M.L., Lin S.P., Batzios S. (2023). A phase I/II study on intracerebroventricular tralesinidase alfa in patients with Sanfilippo syndrome type B. J. Clin. Investig..

[26] Ohsawa M., Kotani M., Tajima Y., Tsuji D., Ishibashi Y., Kuroki A., Itoh K., Watabe K., Sango K., Yamanaka S. (2005). Establishment of immortalized Schwann cells from Sandhoff mice and corrective effect of recombinant human beta-hexosaminidase A on the accumulated GM2 ganglioside. J. Hum. Genet..

[27] Pennybacker M., Liessem B., Moczall H., Tifft C.J., Sandhoff K., Proia R.L. (1996). Identification of Domains in Human Beta-Hexosaminidase That Determine Substrate Specificity. J. Biol. Chem..

[28] Akeboshi H., Chiba Y., Kasahara Y., Takashiba M., Takaoka Y., Ohsawa M., Tajima Y., Kawashima I., Tsuji D., Itoh K. (2007). Production of recombinant beta-hexosaminidase A, a potential therapeutic enzyme for Tay-Sachs and Sandhoff diseases, in the methylotrophic yeast *Ogataea minuta*. Appl. Environ. Microbiol..

[29] Akeboshi H., Kasahara Y., Tsuji D., Itoh K., Sakuraba H., Chiba Y., Jigami Y. (2009). Production of human beta-hexosaminidase A with highly phosphorylated N-glycans by the overexpression of the *Ogataea minuta* MNN4 gene. Glycobiology.

[30] Duarte V.M., Tibavija S.S., Rojase H.Y.T., Leal A.F., Zamora-Moreno S., Bojaca J.A., Alméciga-Díaz C.J., Espejo A.J. (2023). Evaluation of hydrolytic activity of two recombinant N-acetylglucosaminidases as potential therapeutic tools for mucopolysaccharidosis type IIIB. Mol. Genet. Metab..

[31] Regier D.S., Proia R.L., D’azzo A., Tifft C.J. (2016). The GM1 and GM2 Gangliosidoses: Natural History and Progress toward Therapy. Pediatr. Endocrinol. Rev. PER.

