# Supplementary material for: Yeast-Produced Human Recombinant Lysosomal β-Hexosaminidase Efficiently Rescues GM2 Ganglioside Accumulation in Tay–Sachs Disease
Source: J Pers Med. 2025 May 10;15(5):196. doi: 10.3390/jpm15050196 (PMC12113087; doi:10.3390/jpm15050196)
Supplement: Supplementary file 1 [file jpm-15-00196-s001.zip › Fig. S1.pdf]

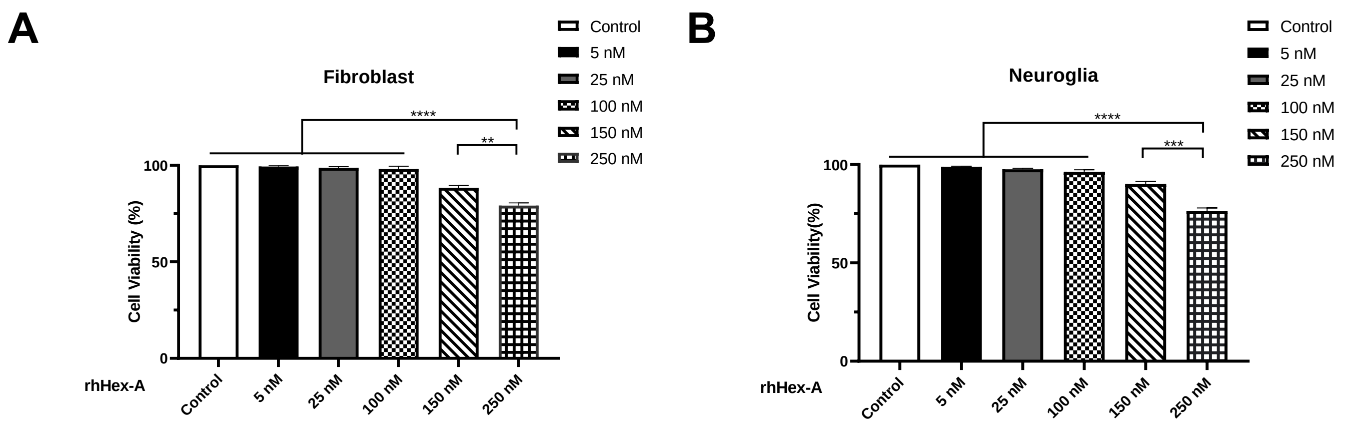

S1

Fig. S1: Cell viability assessed by MTT assay. WT fibroblasts (A) and neuroglia (B) cells were treated with 5 nM, 25 nM, 100 nM, 150 nM and 250 nM at the indicated doses for 72 hours. The data are represented as the mean ± S.E.M. One-way ANOVA analysis was used to determine p-values, as GraphPad shows.
