# Supplementary material for: Yeast-Produced Human Recombinant Lysosomal β-Hexosaminidase Efficiently Rescues GM2 Ganglioside Accumulation in Tay–Sachs Disease
Source: J Pers Med. 2025 May 10;15(5):196. doi: 10.3390/jpm15050196 (PMC12113087; doi:10.3390/jpm15050196)
Supplement: Supplementary file 1 [file jpm-15-00196-s001.zip › Fig. S2.pdf]

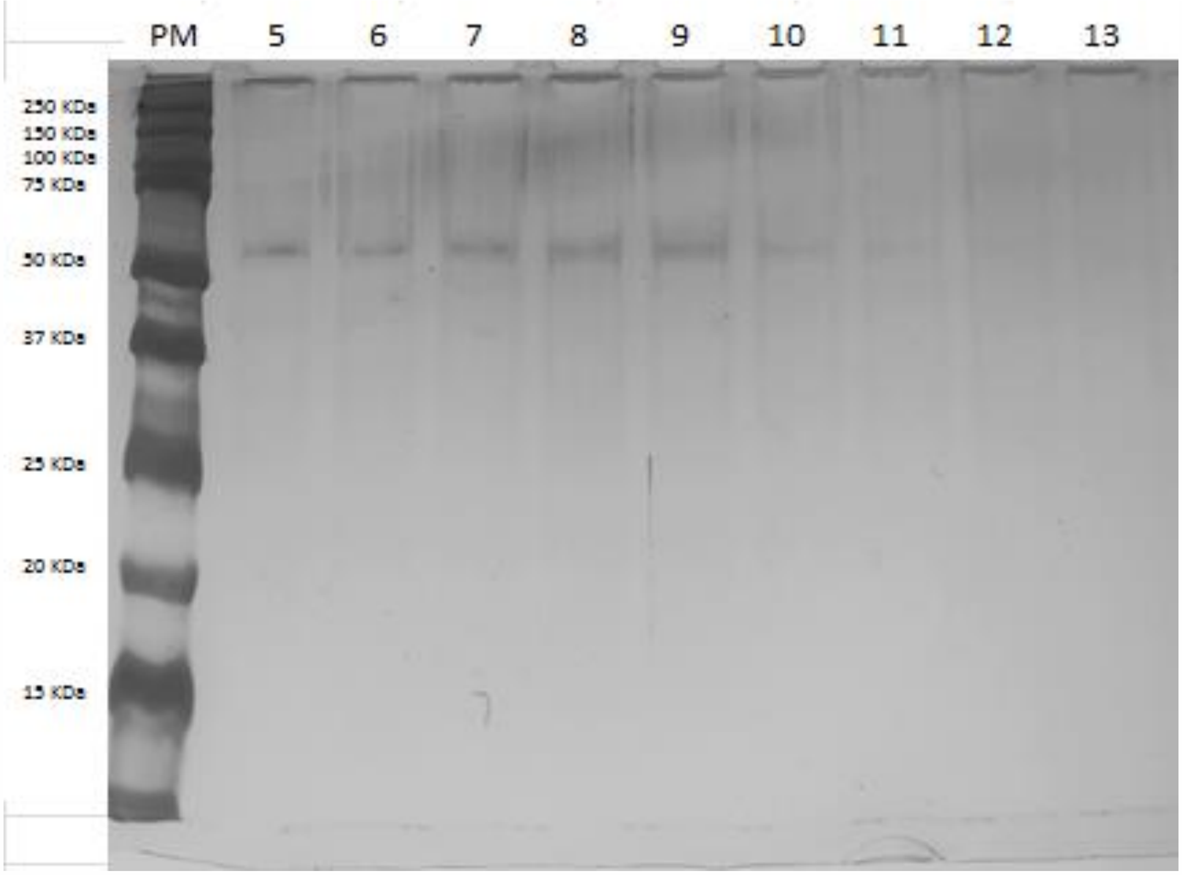

Fig. S2: SDS-PAGE of rhHexA purification. The eluted fractions (5 to 13) obtained during ion exchange chromatography of rhHexA were analyzed by SDS-PAGE under reducing conditions. MW: molecular weight.
